# Supplementary figures and images for: Left Ventricular Ejection Fraction Can Predict Atrial Thrombosis Even in Non-High-Risk Individuals with Atrial Fibrillation
Source: J Clin Med. 2022 Jul 7;11(14):3965. doi: 10.3390/jcm11143965 (PMC9317918; doi:10.3390/jcm11143965)

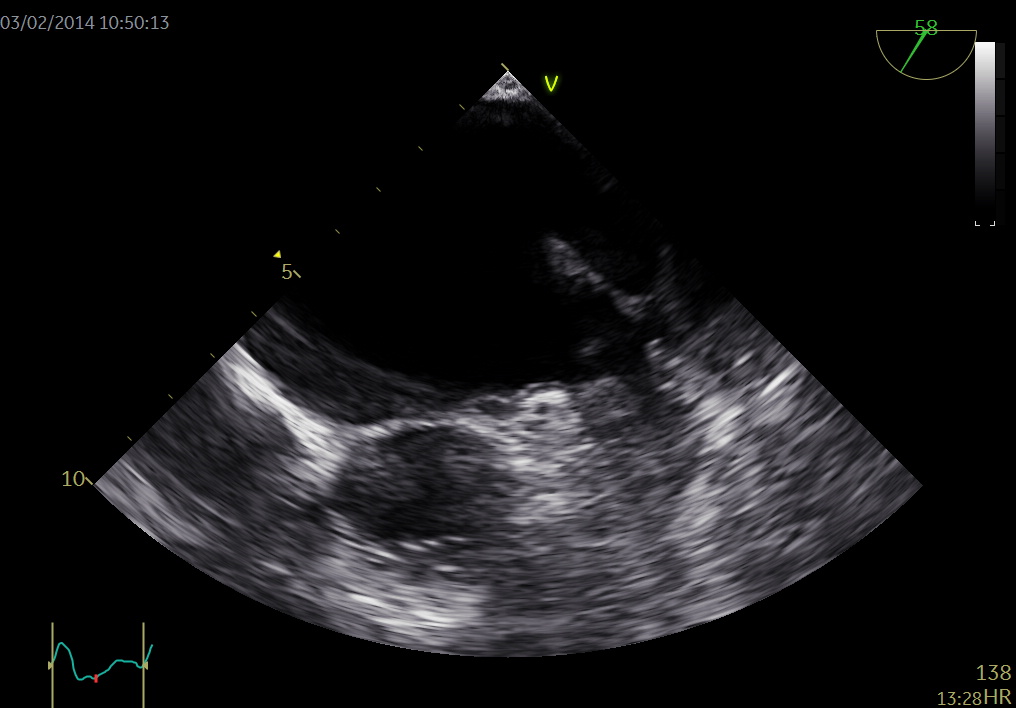

Supplement: Supplementary file 1 [file jcm-11-03965-s001.zip › S1.jpg]

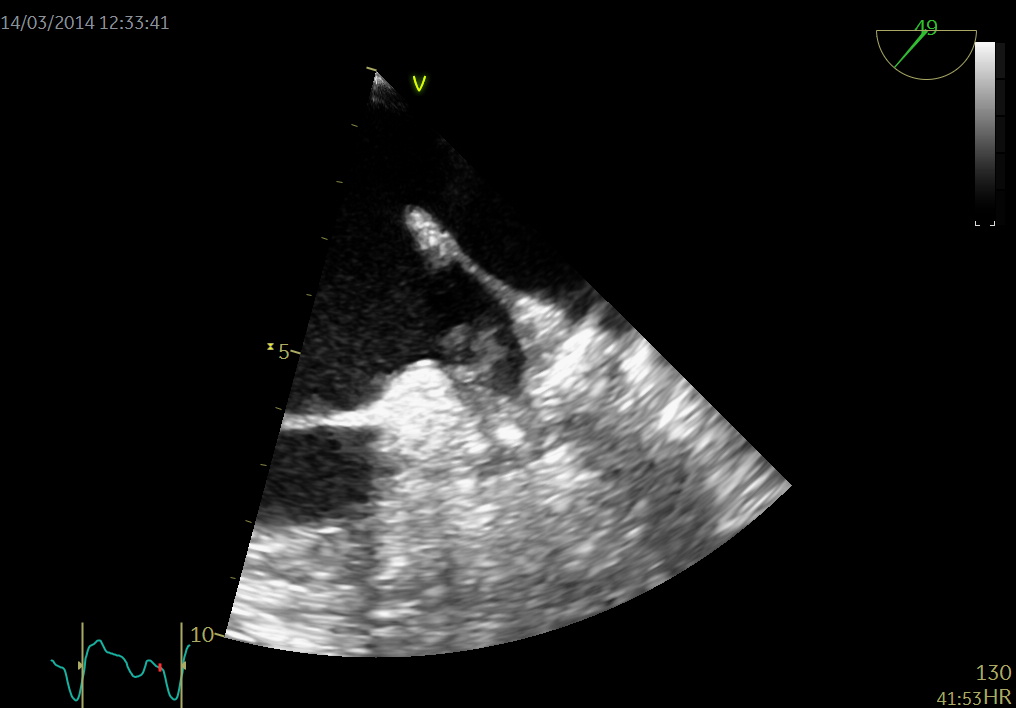

Supplement: Supplementary file 1 [file jcm-11-03965-s001.zip › S2.jpg]

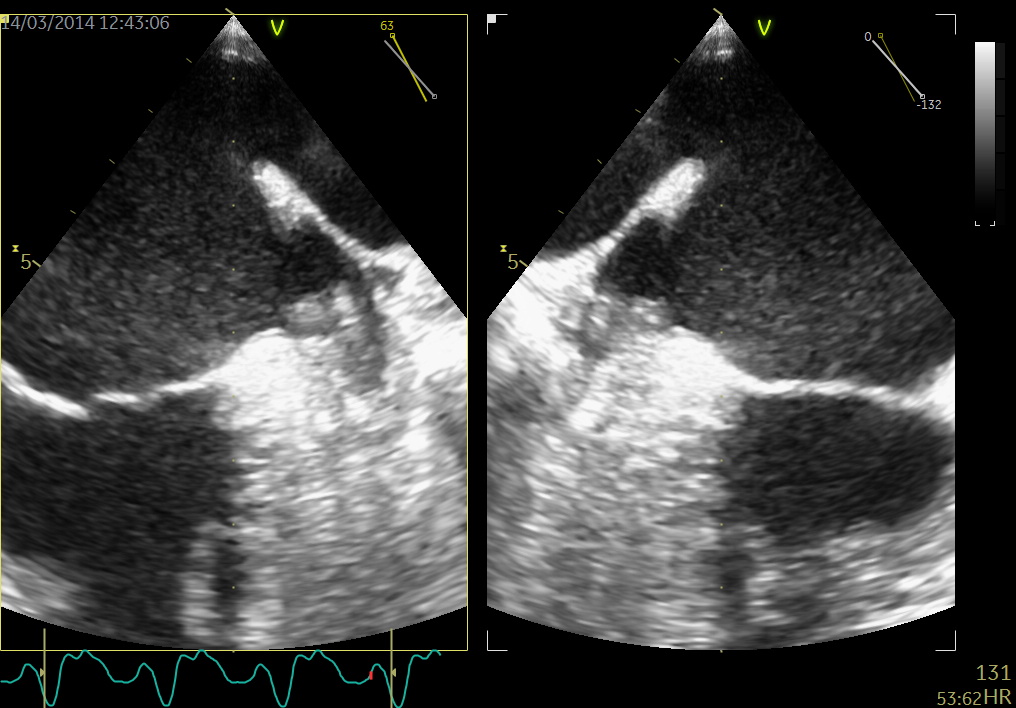

Supplement: Supplementary file 1 [file jcm-11-03965-s001.zip › S3.jpg]

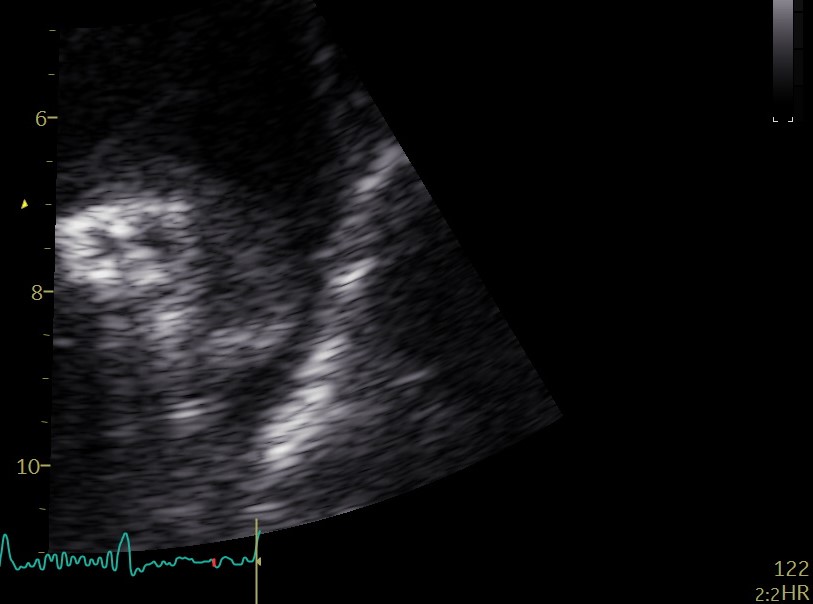

Supplement: Supplementary file 1 [file jcm-11-03965-s001.zip › S4.jpg]

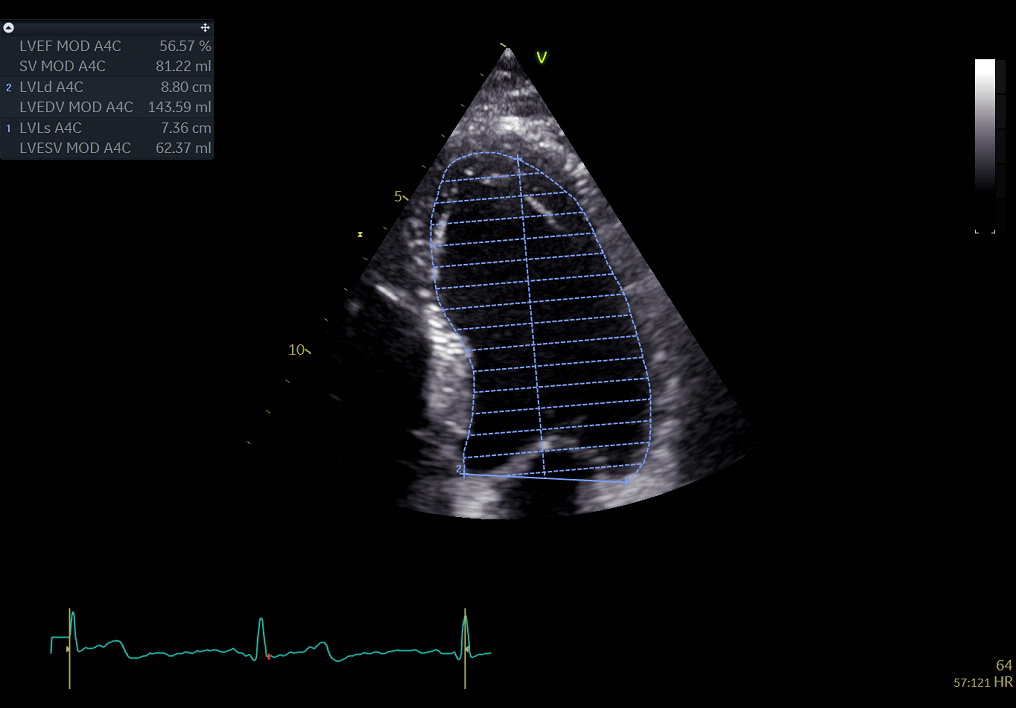

Supplement: Supplementary file 1 [file jcm-11-03965-s001.zip › S5.jpg]

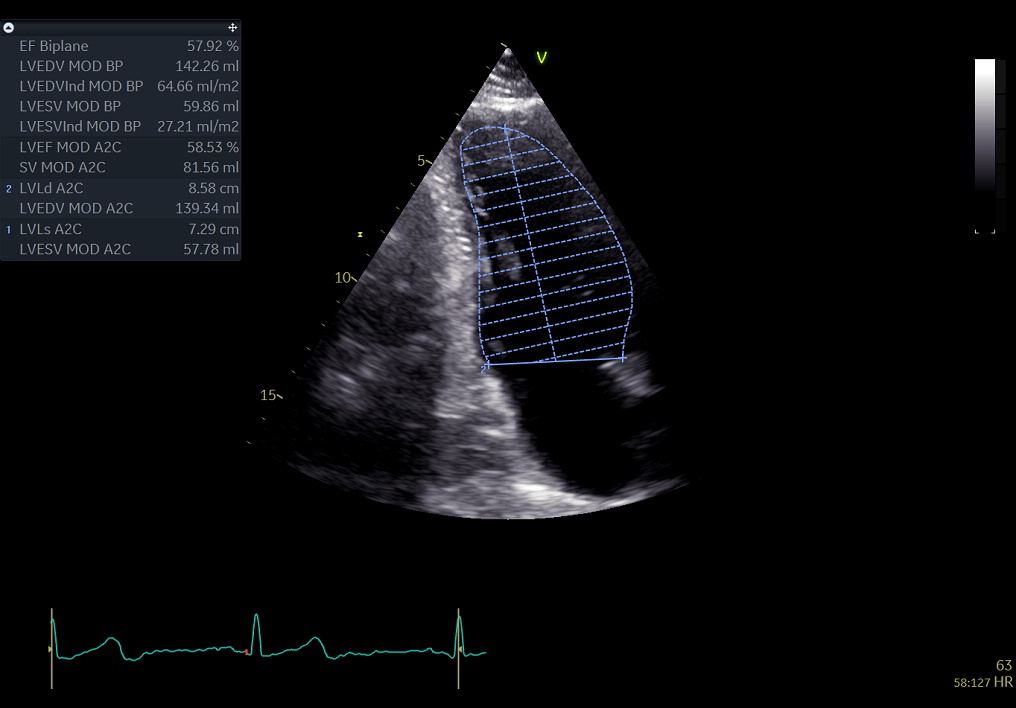

Supplement: Supplementary file 1 [file jcm-11-03965-s001.zip › S6.jpg]

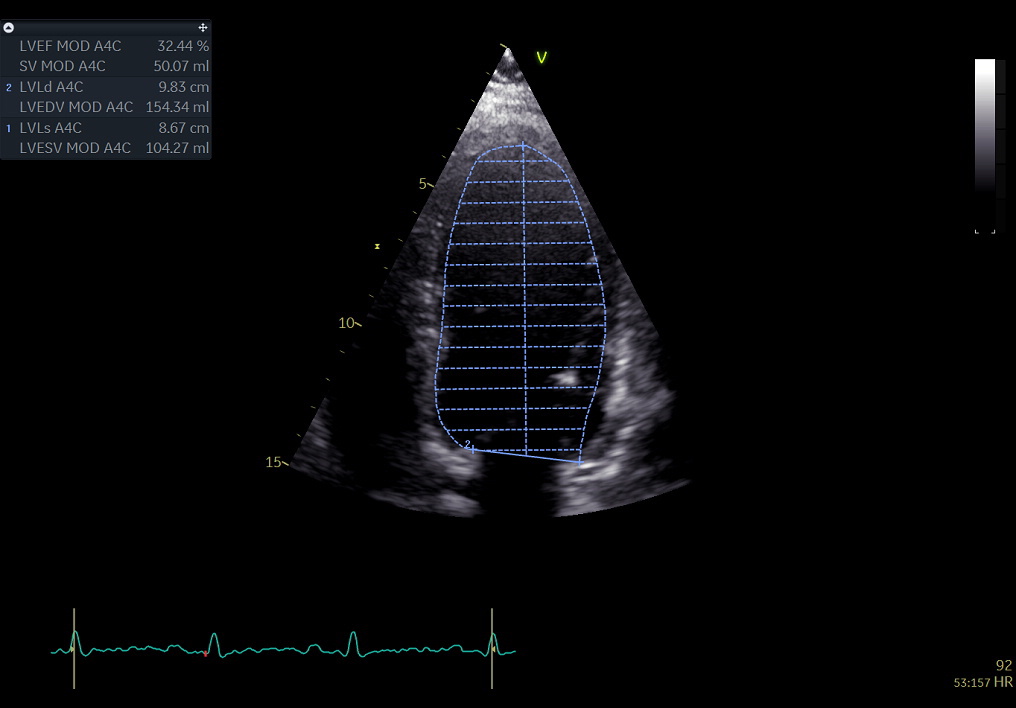

Supplement: Supplementary file 1 [file jcm-11-03965-s001.zip › S7.jpg]

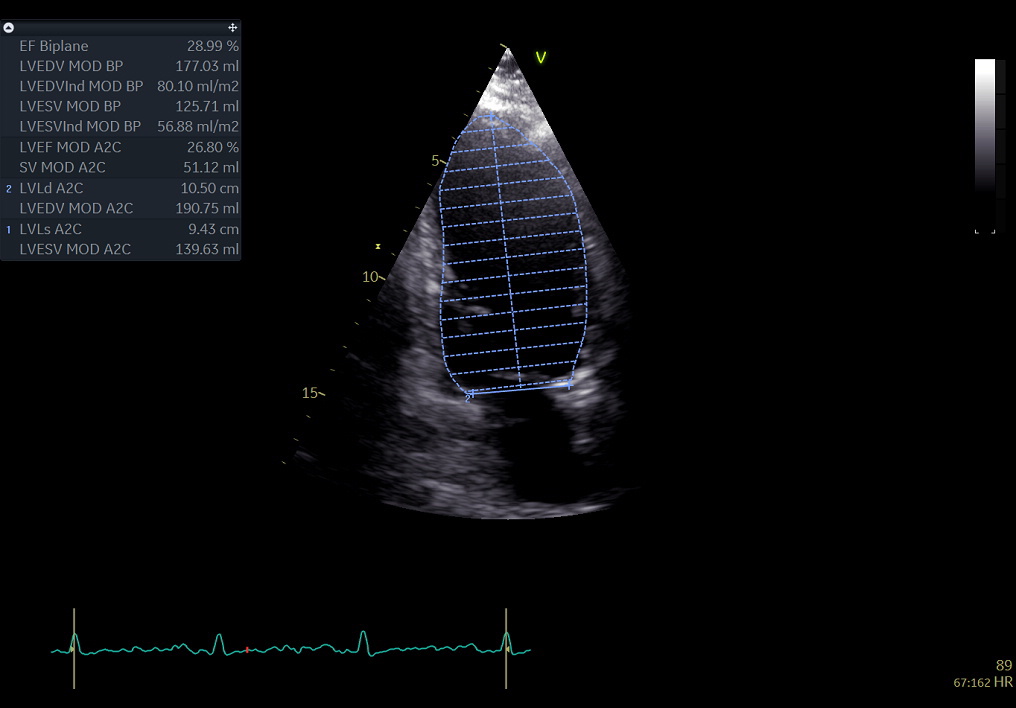

Supplement: Supplementary file 1 [file jcm-11-03965-s001.zip › S8.jpg]
